# Effect of Air-Polishing on 3D Surface Integrity of Composite Dental Restorations—Comparison of Three Different Powders with Reduced Abrasiveness

**DOI:** 10.3390/ma19010140

**Published:** 2025-12-31

**Authors:** Joanna Janiszewska-Olszowska, Agnieszka Droździk, Katarzyna Tandecka, Katarzyna Grocholewicz

**Affiliations:** 1 Department of Interdisciplinary Dentistry, Pomeranian Medical University in Szczecin, al. Powstancow Wlkp. 72, 70-111 Szczecin, Poland; jjo@pum.edu.pl (J.J.-O.); katarzyna.grocholewicz@pum.edu.pl (K.G.); 2 Laboratory of Preclinical Peridontology, Pomeranian Medical University in Szczecin, al. Powstancow Wlkp. 72, 70-111 Szczecin, Poland; agnieszka.drozdzik@pum.edu.pl; 3 Faculty of Mechanical Engineering and Energy, Koszalin University of Technology, ul. Raclawicka 15-17, 75-620 Koszalin, Poland

**Keywords:** air-polishing, surface integrity, sodium bicarbonate, glycine, erythritol, composite

## Abstract

Composite restorations are inevitably exposed to prophylactic procedures associated with a risk of surface damage (loss of substance and roughening). The aim of the present study was to compare the effect of air-polishing with three different powders of reduced abrasiveness on composite fillings. Forty-eight specimens of microhybrid light-cure composite were randomly divided into three groups (n = 16 each), scanned in 3D and air-polished with the following: sodium bicarbonate (40 µm), glycine (25 µm) and erythritol (14 µm), respectively. Then, the specimens were rescanned and the data were processed in specialized 3D analysis software. Loss of composite material was visible in all specimens. The estimated mean composite volume loss was higher for sodium bicarbonate and erythritol (0.09 mm^3^ and 0.08 mm^3^, respectively) than for glycine (0.05 mm^3^). No statistically significant differences were found between sodium bicarbonate and erythritol. Powder particles were additionally characterized from SEM images (N = 1600 per powder), using equivalent circular diameter (ECD) and shape descriptors (aspect ratio and circularity). Therefore, glycine powder should be preferred when the primary goal is minimizing composite abrasion. When higher composite material removal is acceptable, erythritol and sodium bicarbonate may be considered to be interchangeable under the present conditions due to their comparable abrasive effect.

## 1. Introduction

Gingivitis and periodontitis, next to caries, are the most widespread oral diseases initiated by pathogenic microorganisms organized as biofilm. Inflammation in marginal periodontium remains closely related to biofilm that accumulates on teeth and restorations adjacent to the gingival margin. Although genetic and environmental factors contribute to the development of periodontal diseases, mechanical biofilm removal constitutes the golden standard for their prevention and treatment effects maintenance [1,2,3]. Two important elements of this standard involve self-performed daily toothbrushing and professional mechanical plaque removal [2,3]. Besides conventional methods of biofilm removal, such as hand periodontal instruments, ultrasonic scalers and rubber-cup polishing, alternative procedures based on a slurry of abrasive powder, pressurized air and water (air-polishing) can be applied [4,5,6].

Despite being an effective method, an air-polishing performed periodically with abrasive powders may result in tooth substance loss [7,8,9,10], as well as restoration wear and surface alteration [11,12,13].

The potential damage may be considered in two aspects: loss of substance (dental hard tissue or filling) and surface alteration (increase in roughness). On the other side, the smooth, polished surface of composite restorations facing gingival tissue and those placed beneath the gingival margin are crucial for gingival health.

High abrasive properties of the earliest commercially available powders and potential risk of soft and hard tissue damage have forced introduction to the market of less abrasive powders [10,14]. A reduction in grain size and hardness allowed for new types of powders based on glycine and erythritol to be applied near and below the gingival margin [4,14,15,16]. Furthermore, sodium bicarbonate-based powder has been modified, resulting in grain size reduction, which potentially decreased abrasiveness.

Glycine-based powder has a lower abrasive effect in comparison with sodium bicarbonate [9,10,12]. This powder was demonstrated to be effective in biofilm removal and safe for both tooth structure and restorative materials [17]. An erythritol-based air-polishing powder was introduced in 2003 [10]. Similarly to glycine, erythritol-based powder is considered safe for the enamel surface and suitable for regular cleaning [18] and is recommended for use during periodontal prophylaxis, treatment and maintenance.

There is data available in the literature showing the effect of air-polishing with sodium bicarbonate and glycine on a resin composite restoration surface [10,11,12,17]. However, information about the possible composite surface alteration caused by erythritol is scarce and refers to roughness compared to other powders [19] and a comparison with a polishing paste [20]. No studies have been found comparing the loss of composite resulting from air-polishing with erythritol compared to other powders. Nevertheless, it has been proven that even powders of a reduced abrasiveness (sodium bicarbonate 40 µm, glycine 25 µm and erythritol 14 µm) caused a statistically significant increase in the surface roughness of a composite material [19].

As composite restorations of increased roughness and surface wear promote bacterial adhesion and biofilm formation, it is reasonable to evaluate the local effects of air-polishing with erythritol compared to other air-polishing powders on restoration surface integrity. The aim of this in vitro study was to evaluate the substance loss of microhybrid restorative composite restorations resulting from air-polishing with erythritol/chlorhexidine powder in comparison to glycine and sodium bicarbonate with reduced mean sizes of the particles, e.g., to compare three different air-polishing powders in terms of their effect on the integrity of a microhybrid restorative composite material.

A mechanistic description of the abrasive process at work during air-polishing is a key requirement for a better understanding of the varying behaviors of differing powders. Abrasion occurs through the kinetic energy of the particle-laden air–water jet, interacting and moving over the composite surface. The intensity and manner of the surface interactions are determined by interrelated variables such as particle hardness, particle size and particle shape, which are defined by angularity and elongation or aspect ratios. More angular and faceted particles could potentially increase contact pressures and involve micro-ploughing and micro-cutting mechanisms, whereas a more equant particle size composition with a greater fraction of smaller and less faceted powder could potentially indicate a predominant deformation process under impact or a micro-peening-like mechanism. The size of the particulates may also play a significant role in determining the particle access and penetration ability of the air–water jet towards the microscopic detail on the surfaces. Thus, besides the bulk measurement of material removed from the composite surfaces, this work uses scanning electron microscopic (SEM) analysis and a description of the used powder materials by determining the size and shape parameterization defined by the aspect ratios and circularity indices.

## 2. Materials and Methods

Forty-eight specimens of a microhybrid light-cure composite (Charisma Classic, Kulzer, Hanau, Germany) were prepared by placing the material in incisor-shaped polyester crown forms. The composite was handled and polymerized according to the manufacturer’s instructions. A universal shade (A2, A3) of material was applied in thin layers (max 2 mm), carefully adopted to the crown walls and polymerized using an LED curing light (T-led, SternWeber, Imola, Italy) for 40 s from each side with the light tip approximately 1 mm away from the specimens for each site. The irradiance of the curing light in Standard mode with an 8 mm light guide was 1400 mW/cm^2^ (manufacturer’s specification; ±20%). After being cured, the material received no finishing treatment. The specimens were numbered in sequence and scanned using a 3D optical scanner (Atos III, Triple Scan, GOM, Braunschweig, Germany), using a lens of the field of 170 × 130 × 130 mm. Optical scanning by Atos is based on the technique of triangulation: two cameras observe the stripes projected on the object. The camera sensor point’s coordinates are calculated for each pixel with high precision. Subsequently, the resulting virtual elements are transformed into reference elements. The precision of the scanner is maintained by a regular calibration procedure; thus, error study is not needed.

**Figure 1:**
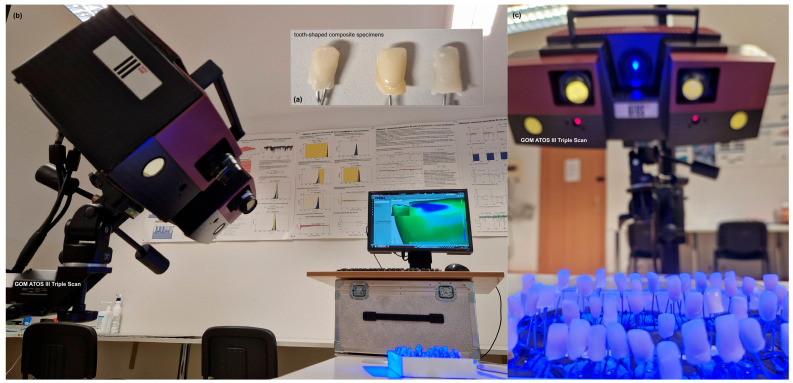
Measurement arrangement for the analysis of volumetric losses by means of the ATOS III Triple Scan optical 3D scanner (GOM, Braunschweig, Germany). Tooth-shaped samples made of composite (Charisma Classic/microhybrid light-cured composite) are represented during the 3D-measurement process prior to the respective air-polishing: (**a**) view of exemplary tooth-shaped composite specimens; (**b**) side view of the ATOS III Triple Scan measurement system; and (**c**) front view of the ATOS III Triple Scan with the specimens visible during the 3D-scanning procedure.

In the current experimental configuration, each dental composite specimen was scanned prior to undergoing air-polishing procedures in order to provide a baseline model. This method of scanning resulted in the generation of high-density point clouds that typically included tens of thousands of points per specimen. Examples of photographs of the dental composites prior to undergoing the experimental procedure, the ATOS III Triple Scan system used in the study, are included in Figure 1.

Sample-size calculation has been performed in order to verify the sample size, using the mean values and standard deviations for the composite volume loss and a power of 0.8 and the formula for sample size calculation in ANOVA, yielding 15 specimens for each of the groups to be compared.

Then, the prepared specimens were randomly divided into three groups of sixteen specimens each. Each air-polishing procedure was performed once per specimen, yielding 16 independent repetitions for each powder type. The cervical areas were subjected to air-polishing using a standard unit (Air-Flow Master, EMS SA, Nyon, Switzerland) with three different powder chambers for variant air-polishing powders, based on the following: sodium bicarbonate—Air-Flow Supragingival Comfort Classic with an average grain size of 40 µm (group SB); glycine—Air-Flow Subgingival Perio with an average grain size of 25 µm (group G); and erythritol—Air-Flow Sub + Supragingival Plus with an average grain size of 14 µm (group E), all from EMS SA, Nyon, Switzerland (The average grain sizes of the powders were provided by the manufacturer). An important aspect of the present study is that the three powders investigated (glycine, erythritol and sodium bicarbonate) differ not only in their chemical composition but also in their particle size distribution. The particle size of the abrasive agent has a direct influence on the way energy is transferred to the composite surface and, consequently, on the resulting wear pattern. However, the materials used in the present study are all commercially available. They are not produced in uniform particle sizes; thus, in routine practice, clinicians have to choose between powders that differ in this aspect. Thus, the present study reflects the characteristics of the materials as they are actually marketed, provided to clinicians and applied in daily practice. We acknowledge that this factor limits the extent to which the effect of particle size alone can be isolated, but we consider the inclusion of powders available on the market in their original form as being essential for preserving the applicability of the results.

The powder chamber was refilled after each air-polishing run to ensure maximum reproducibility of the powder emission. The air-polishing procedure was performed by an experienced specialist in periodontology, using the standard unit according to the regular clinical procedure with a constantly sweeping movement and the settings recommended by manufacturer at the pressure of 2.5 bar at a spraying distance of 3 mm, at an angle between the nozzle and specimen surface of 45° and with a spraying time of 5 s with a countdown via electronic control of the device.

Then, the specimens were rescanned and alteration of the macrogeometric features of the specimen resulting from air-polishing were analyzed using GOM Inspect software v7.5 (GOM, Braunschweig, Germany), allowing us to inspect the digitalized models measured using Atos Triple Scan with reference models. The references for comparisons were scans of the pretreatment surfaces; superimposition and comparison proceeded, using the specimen before air-polishing as reference and the specimen after air-polishing as virtual objects. GOM Inspect software allowed us to calculate the defect depth and volume loss. Every point of the nominal data has been compared with the reference data, thus calculating the shape alteration of the entire analyzed object. This analysis enabled us to calculate the composite volume loss and depths in different locations of the analyzed surface.

The level of significance (α) was established at 0.05. Seven univariate linear regression models were developed and implemented, with each employing a robust estimator to determine the effects of the different types of air powder on each variable describing composite loss. The variances in the outcome metrics among the different air powders were methodically quantified by examining the estimated marginal means (EMMs) derived from the regression models. These EMMs represented the average expected outcomes associated with each air powder type, as per the model. Pairwise comparisons were made of the EMMs, corresponding to each air powder type. In order to account for multiple comparisons and bolster the reliability of the findings, the Šidák correction was applied. The accuracy of these estimates, which was indicative of the variability in the sample data, was measured by the standard error (SE). The significance of the findings was evaluated using the z statistics. The practical significance of these results was contextualized through the calculation of Cohen’s d. Analyses were conducted using the R Statistical language (version 4.3.1; R Core Team, 2023) on Windows 10 × 64 (build 19045).

The morphology of particles was analyzed using scanning electron microscopy images acquired using a Phenom ProX tabletop electron microscope (Phenom-World BV, Eindhoven, The Netherlands). The powders were placed onto SEM sample stubs using adhesive carbon tape. The powders were carefully spread to reduce any overlap or agglomeration of particles to minimize overlap to ensure that the boundaries between individual particles remained visible within a two-dimensional projection. The SEM images acquired were then analyzed for particle morphology using MountainsLab^®^ version 11 software (Digital Surf, Besançon, France). A total of 1600 particles for each powder type were analyzed (the first 1600 particles detected within each dataset), ensuring that an equal sample size for each powder type could be directly compared. The two-dimensional images acquired using SEM analysis allowed for particle segmentation to take place. The particle masks generated could then be used to calculate size- and shape-related descriptors for each particle.

A measure that described the particle size was the equivalent circle diameter (ECD). The ECD can be defined as the diameter of the circle that has the same area as the two-dimensional projected area of the particle. If A represents the area of the particle, then ECD can be calculated using the following equation:

$$ECD=2\sqrt{\frac{A}{\pi }}$$

This provides a strong estimation of the particle size of irregularly shaped objects because it is calculated on the basis of the area and is not affected by the largest dimension. The aspect ratio of the particles was measured from the orthogonal dimensions obtained from the two-dimensional projection of the particles, which are commonly measured by the Feret diameters. The maximum Feret diameter, Fmax, is the longest distance between two parallel tangents to the particle shape, and the minimum Feret diameter, Fmin, is the shortest distance between two parallel tangents to the particle shape. Thus, the aspect ratio measurement is often calculated from the ratio of these two lengths:

$$Aspect\;ratio=\frac{Fmax}{Fmin}$$

Particle circularity (Circ) was used as a shorthand metric that characterized the extent to which a particle’s shape approached a perfect circle and the degree of departure of its boundary from a rounded shape. The equation for circularity is given by the following (particle area A and perimeter P):

$$Circ=\frac{4\pi A}{P^{2}}$$

For all descriptors—equivalent circular diameter (ECD), aspect ratio and circularity—results were calculated for each individual particle and then summarized using descriptive statistics. For ECD, the arithmetic mean $\overset{-}{x}$ sample standard deviation (SD), minimum value, maximum value and empirical percentiles D10, D50 and D90 were determined, where D50 represents the median of the distribution, for N = 1600 particles per powder. For the aspect ratio and circularity, median values of robust distributions were given together with first and third quartiles, expressed as median [Q1–Q3]. Additionally, for circularity, the min–max range was also given. In an attempt to provide an intuitive visualization of particle size distributions, ECDF plots of ECD were used to facilitate a direct comparison of entire size distributions among different powders, while for showing the distributions of shape descriptors (shape ratio, circularity), box-plots were used to provide a compact representation of distributions around central tendencies and dispersions.

## 3. Results

All the newly formed composite samples were first scanned by the ATOS III Triple Scan optical system to provide high-resolution 3D data for baseline 3D models. After the initial data acquisition process, each sample was then treated by the air-polishing process according to the assigned experimental group. Following the immediate completion of the air-polishing process for each sample, the 3D surface models prior to the procedure were superimposed on the 3D surface models acquired post-air-polishing. By means of the best fit alignment algorithm, the point-to-point analysis of the data was made possible. Based on the aligned 3D models obtained from the previous process, dense deviation maps were developed. These maps quantified the aforementioned parameters in the context of the material lost by the powder used in the air-polishing process by directly correlating the local values to the lost material. Printscreens from typical superimpositions of the scans have been presented in Table 1.

Some loss of the composite material was visible in all the analyzed specimens. The depths of the resulting defects, the total volume of the composite loss, the area affected and the mean volume of loss per unit of area affected for groups SB, G and E have been presented in Table 2. Descriptive raw means are presented in Table 2, whereas statistical comparisons are based on EMMs (Table 3).

**Figure 2:**
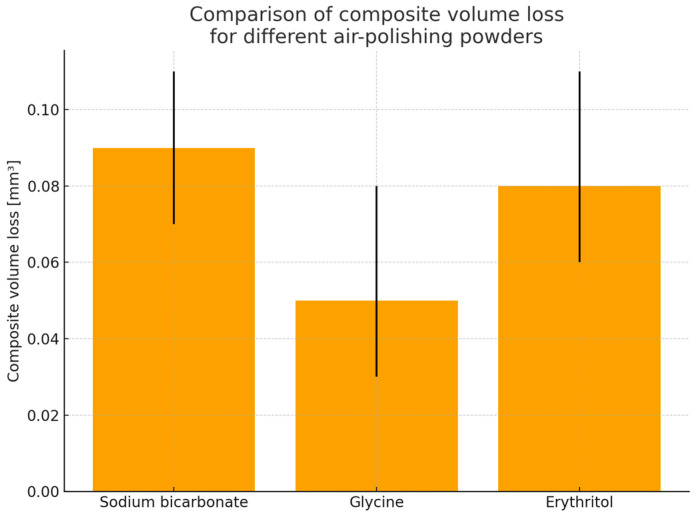
Average composite volume loss [mm^3^] after air-polishing using three low-abrasive powders: sodium bicarbonate, glycine and erythritol.

The estimated marginal mean and confidence intervals of the mean defect depth, maximum defect depth, composite volume lost, area affected and volume lost per unit of area affected have been presented in Table 3 and Figure 2.

The estimated marginal means (EMM) indicate that both erythritol and sodium bicarbonate lead to a higher depth of composite loss, with EMM values of 3.81 µm and 3.68 µm, respectively, compared to glycine, which exhibits a notably lower EMM of 2.66 µm, indicating its milder abrasive effect.

A comparative analysis across air powders referring to mean defect depth, maximum defect depth, composite volume lost, area affected and volume lost per unit of area affected has been presented in Table 4.

The significant positive estimate of 1.02 µm between sodium bicarbonate and glycine, coupled with a large effect size (Cohen’s d = 0.85), underscores the more pronounced abrasive capability of sodium bicarbonate. Conversely, the comparison between sodium bicarbonate and erythritol shows an insignificant difference in mean depth, as evidenced by the near-zero estimate and a negligible effect size, indicating similar abrasive properties between these two powders.

Furthermore, the contrast between glycine and erythritol, which yielded a negative estimate of −1.15 µm with a large effect size (Cohen’s d = −0.96), strongly points to erythritol’s higher abrasiveness compared to glycine. This is substantiated by the statistical significance (*p* = 0.010) of this result.

It is visible that a substantial amount of composite material is removed by each of the air-polishing powders. The depth and area of damage and volume composite loss were different for individual specimens.

The results of the EMMs showed that the average defect depth after air-polishing was lowest for glycine (2.66 μm) and larger for both erythritol and sodium bicarbonate (3.81 μm and 3.68 μm, respectively) (Table 3 and Figure 2). Pairwise comparisons confirmed a significantly larger average depth for sodium bicarbonate compared with glycine (estimate 1.02 μm, *p* = 0.028), and for erythritol compared with glycine (estimate −1.15 μm for glycine–erythritol contrast, *p* = 0.010), and not significant between sodium bicarbonate and erythritol (*p* = 0.598) (Table 4). These results, in combination with comparisons for maximum depth, total volume loss, affected area and volume loss per unit area, communicate a common message: glycine represents the most conservative surface parameter under the current testing conditions, while both erythritol and sodium bicarbonate produce a comparable composite material loss.

**Figure 3:**
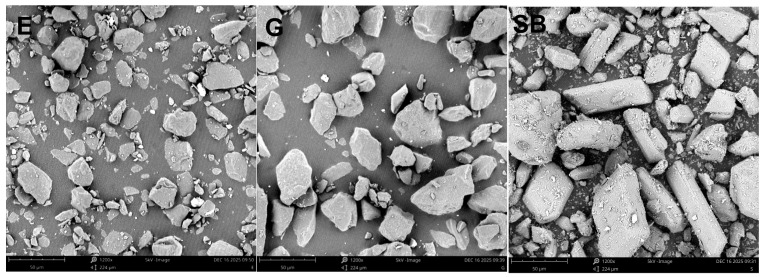
Representative SEM micrographs of the air-polishing powders: erythritol (E), glycine (G) and sodium bicarbonate (SB). Images were acquired using a Phenom ProX tabletop electron microscope (Phenom-World BV, Eindhoven, The Netherlands).

The descriptors for the powders themselves were the size and morphology descriptors, which were measured from SEM (Figure 3) images and calculated for N = 1600 particles for each powder (Table 5).

**Figure 4:**
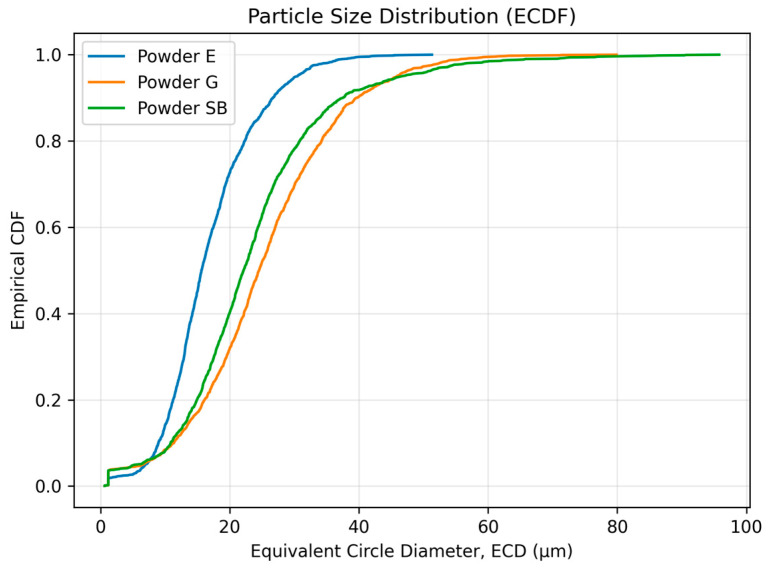
Empirical cumulative distribution function (ECDF) of the equivalent circle diameter (ECD) for the three powders: erythritol (E), glycine (G) and sodium bicarbonate (SB) (N = 1600 particles per powder).

**Figure 5:**
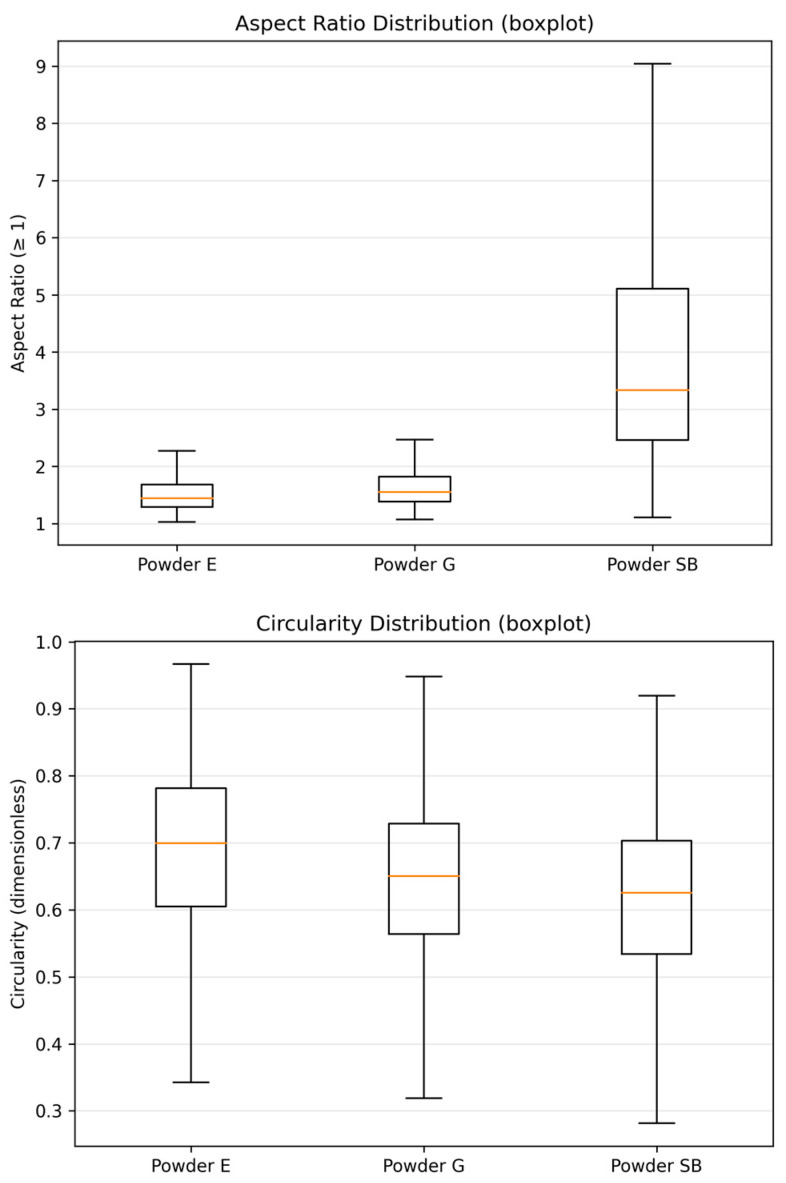
Boxplots of particle shape descriptors for the three powders: (top) aspect ratio and (bottom) circularity (Circ) for erythritol (E), glycine (G) and sodium bicarbonate (SB) (N = 1600 particles per powder).

The powder with the smallest size distribution was erythritol (E), with mean ECD = 16.86 ± 7.23 µm and D50 = 15.64 µm, while the particles were larger for glycine (G), with mean ECD = 25.22 ± 11.60 µm and D50 = 24.55 µm. The mean ECD for sodium bicarbonate powder was (SB) = 23.65 ± 12.42 µm with a smaller D50 = 22.15 µm. However, this powder exhibited the widest size range and the most pronounced upper-tail extension, with the maximum ECD reaching 95.79 µm. The empirical cumulative distribution function (ECDF) plot of the equivalent circle diameter (ECD) provides a clear visualization of these differences in particle size distributions (Figure 4), where the ECDF for the erythritol powder is shifted downwards compared with the other two. In terms of morphology descriptors for shape characteristics, in this study, we show that SB has higher anisotropy than E and G. The median aspect ratios for SB = 3.333 [2.458–5.104] compared with E = 1.439 [1.290–1.683] and G =1.551 [1.384–1.818] (Table 5) are consistent with the aspect ratio box-plot results shown in Figure 5. The reverse is true for circularity. Here, the highest median circularity = E = 0.699 [0.605–0.782], followed by G = 0.650 [0.564–0.729] and the least is SB = 0.625 [0.534–0.703], with the longest range indicating highly non-circular particles for all three powders, as shown in Table 5 and Figure 5. Based on the results for the descriptors for the powders themselves, the powders did not only differ in size, size range and shape characteristics: the results show that SB has a substantially larger aspect ratio compared with E and G.

On the contrary, circularity displayed a reverse order: E displayed a high median circularity value of 0.699 [0.605–0.782] compared with G and SB, which stood at 0.650 [0.564–0.729] and 0.625 [0.534–0.703], respectively (Table 5 and Figure 5). The minimum–maximum differences for all powders clearly indicated that highly non-circularly shaped particulate matter was also dispersed in all powder samples (Table 5), and the lowest circularity measurements were also displayed by G and SB (Figure 5). All morphological parameters clearly indicated that not only did E and G differ in particle size and size distribution width, but a notable variation also persisted in the powder particle morphology, where highly elongated and non-circular particulate matter in SB differed substantially compared with E and G.

## 4. Discussion

This is the first study showing the effect of air-polishing with erythritol-based air-polishing powder on the surface integrity of composite fillings in comparison to other powders of a reduced abrasiveness. The use of a precise 3D scanner, superimpositions of the scans and a specialized software has allowed for objective quantitative measuring of 3D geometric alterations of the surface.

Restorations in the cervical region might stain over time and then are subjected to air-polishing, aiming to remove the discoloration. Nevertheless, cervical restorations are inevitably exposed to prophylactic procedures. This implies a risk of surface damage, including the loss of substance and roughening. The loss of integrity of the composite filling material may result in plaque accumulation, marginal leakage and secondary caries. It is considered that an increase in surface roughness above the Ra threshold of 0.2 µm increases the available area for adhesion of pathogenic microorganisms, thus promoting biofilm formation [21]. This can enhance composite degradation by bacterial esterases, thereby further increasing the surface roughness of restorations [22,23]. Moreover, it should be kept in mind that although air-polishing devices are used to remove supra- and subgingival staining, air-polished composites are more susceptible to superficial staining from coffee beverages; thus, re-polishing after air-polishing could be considered [24].

Scientific evidence exists on the influence of air-polishing on dental tissues. It is obvious that surface damage resulting from kinetic abrasion depends on the size, hardness and angularity of abrasive particles [25]. In general, particles of a rounded shape cause less surface roughening than those with irregular edges [26]. It should be kept in mind that the Mohs hardness of sodium bicarbonate is 2.8, whereas glycine and erythritol rank at 2 [27,28]. Thus, a reduction in particle size and hardness is generally associated with a reduced abrasive potential for dental tissues, although the overall effect depends on multiple coupled factors, including particle morphology and operating parameters. Sahrmann et al. [28], in their micro-CT study of root surface damage, found that glycine-based powder caused a significantly lower abrasion compared to bicarbonate.

According to Bühler et al. [29], defect depths and volume of the dental hard tissue caused by sodium bicarbonate or calcium carbonate powders were significantly greater compared to powders consisting of glycine. The soft tissue modifications using different modes of instrumentation were assessed in publications. The data demonstrate less potential for harm to the gingiva after spraying with glycine powder compared to sodium bicarbonate powder or instrumentation with curettes. Referring to erythritol, Guma et al. [30] found a lesser roughening effect compared to sodium bicarbonate 65 µm on sound enamel, whereas no difference was apparent on artificially demineralized enamel.

The evidence referring to the influence of air-polishing on composites is scarce. All previous studies found, none of which are older than 15 years, have been presented in Table 6. No studies could be found comparing the loss of the composite material between erythritol powder and other air-polishing powders.

During air-polishing, the resin matrix is selectively removed and filler particles are exposed, resulting in surface roughening. A study assessing composite microroughness following air-polishing [19] revealed that the composite surface resulting from air-polishing with 40 µm sodium bicarbonate was characterized by a higher roughness compared to glycine (25 µm) and erythritol (14 µm).

The study by Reinhart et al. [20] compared the roughness of a nanocomposite resulting from repeated air-polishing with erythritol to that resulting from repeated polishing with Prophy Cleanic Paste (no comparisons were made with other air-polishing powders) and revealed a higher roughness after the use of prophy paste. The paste used was of 21 RDA with abrasive particles of perlite—a volcanic glass, containing mainly silicone dioxide and aluminum dioxide and characterized by a Mohs hardness between 5.5 and 7, whereas the hardness of erythritol is 2. The methods used by Reinhart et al. [20], who have tried to simulate the use of air-polishing vs. polishing paste in a rubber cup for 10 years, are different from the present study, as the setting of the present study simulated a single prophylaxis procedure; thus, the results cannot be compared to the present investigation. According to the study by Reinhart et al. 2022 [20], treatment with an erythritol powder, AirFlow^®^Plus, leads to less abrasion and surface roughness of nanocomposite, Ceram X Spectra™ ST, Dentsply restorative material compared to Cleanic^®^ prophy-paste in ProCup Hard (Kerr). The results of the cited study suggest lesser composite damage from erythritol powder compared to prophy paste used in a rubber cup with a force of 1.5 N.

However, a single cleaning procedure with prophy paste did not cause a noticeable loss of substance or roughening of human enamel [7], contrary to air-polishing with erythritol.

According to Barnes et al. [32], glycine powder produces the least damage to the composite material compared to sodium bicarbonate 74 µm, aluminum trihydroxide 80–325 µm, calcium carbonate 55 µm and calcium sodium phosphosilicate (novamin) 25–120 µm, which is consistent with the present study. An early study of 2004 [33] proved that aluminum trihydroxide of Mors hardness 4.0 is destructive to dental materials.

In the study by Shimizu et al. [24], referring to the depths of defects at the interface between dentin and composite material, each specimen was air-polished with either sodium bicarbonate (NaHCO_3_ 65) or one of two glycine powders: 25 µm and 65 µm. SEM observation and a measuring microscope were used to measure the mean and maximum depth of the defects. The air polisher was set at angles of 90° to the interface and at 45° to the interface from both the dentin and resin composite sides. Air-polishing with glycine powders produced defects with less depth and volume than NaHCO_3_ powder (*p* < 0.05). The present finding that erythritol 14 µm is not less abrasive than glycine 25 µm is consistent with there being no difference between two different Gly powders (65 µm and 25 µm). This suggests that, for the commercially available powders tested under the present conditions, particle size alone does not fully explain the observed abrasion, and that hardness and particle morphology are likely to contribute substantially. Based on the working principles established in previous studies of air-polishing powders, the three powders used in this study support different levels of surface modification, due to the different relative proportions of impact- to slide-dominated abrasion. Sodium bicarbonate, with a higher hardness value and a larger nominal particle size, promotes pronounced micro-ploughing/micro-cutting and may cause micro-chipping or filler pull-out (not directly assessed in the present study), resulting in substantial material removal. Glycine, with lower hardness and smaller particle size, produces mainly small-scale abrasion with reduced micro-cutting and therefore has the lowest level of material removal. Erythritol, despite smaller particle sizes than glycine, produces material removal that is comparable to sodium bicarbonate rather than glycine. This outcome reflects the combined influence of particle size, particle shape and angularity, access to surface micro-irregularities and particle properties such as friability and agglomeration, rather than particle size alone. Quantitative particle size analysis carried out during this research provides evidence supporting the conclusion that the abrasive action cannot solely or primarily depend on nominal particle size. Although erythritol (E) had the smallest particle size distribution (mean ECD = 16.86 ± 7.23 µm; D50 = 15.64 µm), the amount of composite material abraded was similar to sodium bicarbonate but higher than glycine, as indicated by the EMMs for the mean values of defect depth (E = 3.81 µm; SB = 3.68 µm; G = 2.66 µm) and the corresponding pairwise comparison results (Table 3 and Table 4; Figure 2). This seems to confirm the existence of a non-negligible coarse part of distribution for E (max ECD = 51.31 µm) and points towards a relationship of size distribution, particularly the coarse part, with momentum transport mechanisms and initial localized damage (Table 5; Figure 4). Additionally, the results of shape analysis point towards strong differences in particle shape for each of the powders studied. Sodium bicarbonate (SB) revealed higher values of aspect ratio (AR) for particles, as well as lower values of circularity, compared to E and G (Table 5; Figure 5), which could indicate a higher number of elongated coarse particles and hence higher values for tangential stresses and micro-ploughing/micro-cutting forces during their interaction with a target surface. Contrary to this, erythritol particles appeared to be relatively circular (with median circularity = 0.699 [0.605–0.782]) but had an amount of abraded composite material that was similar to SB, indicating the dominant role of a combination of particle size distribution, particle shape and other properties of each of E, SB and G, specifically their agglomeration/friability, during abrading, within the given conditions of jetting.

It is noteworthy that the air-polishing powder (AIR-FLOW PLUS, EMS Electro Medical Systems, Nyon, Switzerland) consists of 99.7% erythritol and 0.3% chlorhexidine (mass % of active ingredient in the powder) and expresses antimicrobial and antibiofilm activity [34].

It has been proven that a composite surface obtained under a polyester matrix is rich in organic resin and therefore has a lower hardness than after being subjected to finishing procedures [35,36]. It is known that finishing procedures applied to different composites may produce surfaces of various roughness and hardness [36]. Thus, it might be supposed that air-polishing composite resin could potentially influence its hardness. However, no studies concerning the influence of air-polishing on the nanohardness or wear properties of composite resin could be found. It seems that analyzing the effect of air-polishing on the microhardness of a finished composite surface could be the subject of another study.

Pelka et al. [11] have noticed that the amount of substance loss due to air-polishing is dependent on the composite material; more destruction was noted for flowable than for condensable composite materials. Unfortunately, the authors of the cited study, although they provided the brand names of the powders used, did not mention the size particles. It is worth noticing that constant progress in the field of dental prophylaxis leads to modifications in the particle sizes of the powders. Products available on the market are subjected to discrete changes in their names and a reduction in particle size. Thus, it is difficult to find information on a powder used in a study published years ago [36] and compare the results published to those from other studies.

A possible limitation of the present in vitro study results from an attempt to mimic the clinical procedure in a real clinical situation instead of a standardized setting that could eliminate dentist-related variances in handpiece movements.

The present findings provide new knowledge justifying clinical selection of an appropriate air-polishing powder. In cases where the primary goal is minimal abrasion of the composite material, glycine would be the preferred choice. Erythritol and sodium bicarbonate resulted in higher composite material loss than glycine; therefore, when composite restorations are present, their use should be balanced against the increased risk of restoration wear. The choice between erythritol and sodium bicarbonate can be based on the patient’s or clinician’s preference, as their abrasive properties were similar in the present study.

The limitations of the current in vitro study refer to the inability to capture the oral environment perfectly. Additionally, the study only used one type of composite material. However, other materials might react differently to the abrasion effect of the air-polishing treatment. Future studies can consider the study of various materials comparatively. The study can also consider the cyclic effect of prophylactic treatment. Finally, the study can consider incorporating the effect of biofilms to be more representative of the oral environment. Future studies can also consider the clinical protocol of air-polishing to minimize the wear of the materials while maintaining the effectiveness of the treatment. The powders differed simultaneously in particle size, hardness and morphology, which reflects real clinical conditions but does not allow for isolating the effect of a single parameter.

## 5. Conclusions

- This research presents novel quantitative data based on the high-precision volumetric measurement of the abrasive effect of the commonly used low-abrasive air-polishing materials on composite restorative materials. The results showed that the powder that had the most conservative effect was the one that resulted in the greatest preservation of the composite’s integrity. This powder was found to be glycine.
- The amounts of abrasion caused by erythritol and sodium bicarbonate were found to be equivalent, which suggests that, for the powders tested under the present conditions, particle size alone does not necessarily predict their abrasive characteristics. Scanning electron microscopy (SEM) particle characterization analysis (N = 1600 for each powder) showed variation in particle size, distribution width and shape between each of the powders. Sodium bicarbonate was seen to have a higher aspect ratio and a lower circularity than both erythritol and glycine. The observations reported above emphasize the importance of selecting the powder carefully during the prophylactic procedure when there are composite restorations in the vicinity.
- The results must be seen in the context of the limitations of the study: it was an in vitro experiment involving only one type of composite restorative material. Future studies must consider the role of various restorative materials, cyclic challenges involving prophylaxis methods, the effect of biofilm formation and the realities of clinical practices. This will be crucial for the development of advanced clinical practices involving the coexistence of efficient biofilm removal methods and the prolonged longevity of restorative materials.

## Figures and Tables

**Table 1:** Superimpositions of scans of the surfaces made before and after air-polishing.

Group	SB	G	E	Legend of Depths
	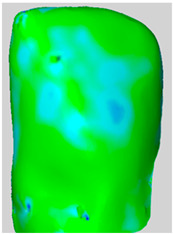	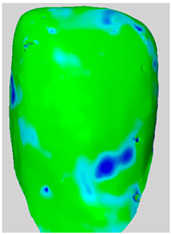	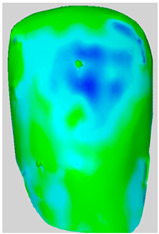	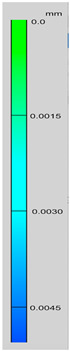

**Table 2:** The depths of the composite defects, volume of composite loss, area affected and volume lost per unit affected.

Group SB Specimen Number	Composite Loss						
	Depth [µm]				Volume [mm^3^]	Area [mm^2^]	Volume Per Unit Area [mm^3^/mm^2^]
	Mean	SD	Max	Min			
1	4.216	0.5962	19.392	1.355	0.191585577	46.56	0.004114811
2	4.017	0.5992	10.127	0.7858	0.180036058	43.15	0.00417233
3	3.316	0.5218	22.69	1.086	0.031945778	9.35	0.003416661
4	3.17	0.5983	16.25	1.505	0.034642474	8.73	0.00396821
5	3.935	0.5583	8.577	0.5964	0.070196152	17.21	0.0040788
6	1.884	0.2305	8.518	1.013	0.032735149	17.81	0.001838021
7	3.62	0.384	13.65	1.794	0.108098293	40.98	0.00263783
8	3.1335	0.497	6.132	1.7314	0.103669555	29.84	0.003474181
9	5.46	0.7357	16.91	1.085	0.12695771	24.23	0.005239691
10	7.19	1.022	18.86	2.99	0.109392243	13.98	0.00782491
11	3.8	0.4264	5.979	1.773	0.070751168	22.81	0.003101761
12	3.53	0.4605	8.199	0.4288	0.093971175	28.68	0.00327654
13	4.056	0.5588	7.326	0.8056	0.072387522	18.43	0.003927701
14	1.715	0.2496	10.17	1.207	0.05365771	31.06	0.00172755
15	6.362	1.148	29.48	3.916	0.139662028	21.91	0.006374351
16	2.328	0.36	6.213	0.6522	0.092185967	37.18	0.00247945
Mean					**0.09449**	**25.74437**	**0.00385**
SD					**0.04827**	**11.73131**	**0.00158**
Max					**0.19159**	**46.56000**	**0.00782**
Min					**0.031946**	**8.730000**	**0.001728**
**Group G** **Specimen number**	**Composite loss**						
	**Depth [µm]**				**Volume [mm^3^]**	**Area** **[mm^2^]**	**Volume per unit area** **[mm^3^/mm^2^]**
	**Mean**	**SD**	**Max**	**Min**			
1	2.575	0.298	4.8226	1.249	0.045144014	22.00	0.002052001
2	2.325	0.3433	8.196	1.29	0.03315761	14.01	0.00236671
3	2.146	0.2832	4.76	0.4185	0.053253381	27.75	0.001919041
4	1.884	0.2613	3.839	0.2613	0.04388874	22.96	0.00191153
5	2.681	0.3036	4.068	0.5681	0.025935801	9.80	0.00264651
6	1.97	0.3357	9.207	0.9491	0.015797062	8.90	0.001774951
7	1.943	0.2679	5.361	1.078	0.023269405	11.76	0.001978691
8	2.709	0.3009	8.885	0.2098	0.036195177	17.71	0.002043771
9	3.813	0.5549	9.554	0.3415	0.100635979	30.14	0.003338951
10	3.247	0.3629	5.991	0.3078	0.087529138	34.95	0.00250441
11	3.06	0.3121	6.46	0.9424	0.08525218	37.83	0.00225356
12	2.925	0.2991	4.448	0.9631	0.042171231	20.51	0.00205613
13	2.182	0.3154	3.895	0.5586	0.033405925	15.56	0.00214691
14	2.856	0.4103	6.786	0.9961	0.09698348	32.04	0.00302695
15	3.98	0.4766	10.6	0.5471	0.129584068	41.55	0.00311875
16	2.405	0.3513	5.962	0.2826	0.02601571	10.40	0.002501511
Mean					**0.0548886813**	**22.366875**	**0.00235252356**
SD					**0.0339557958**	**10.5993432**	**0.000471039575**
Max					**0.129584068**	**41.55**	**0.003338951**
Min					**0.015797062**	**8.9**	**0.001774951**
**Group E** **Specimen number**	**Composite loss**						
	**Depth [µm]**				**Volume [mm^3^]**	**Area** **[mm^2^]**	**Volume per unit area** **[mm^3^/mm^2^]**
	**Mean**	**SD**	**Max**	**Min**			
1	4.49	1.008	17.61	2.502	0.183729179	26.58	0.006912309
2	3.975	0.4342	7.928	0.5168	0.118947668	16.33	0.007283997
3	3.137	0.3791	5.629	0.4735	0.366920822	36.76	0.009981524
4	3.307	0.3297	5.103	0.1818	0.040681361	16.74	0.002430189
5	5.077	0.452	7.751	0.5167	0.130081497	38.53	0.003376109
6	2.355	0.335	4.358	0.8473	0.064215606	25.91	0.00247841
7	2.698	0.3937	8.217	0.5867	0.12644239	49.93	0.002532393
8	4.481	0.5303	7.77	0.525	0.073163292	14.69	0.004980483
9	5.852	0.5809	8.611	0.5323	0.055368108	13.17	0.004204108
10	2.971	0.3511	8.654	1.091	0.046374874	19.21	0.002414101
11	3.414	0.2328	7.354	0.9759	0.034845036	22.88	0.001522947
12	5.254	0.8124	13.97	0.4758	0.185893895	34.98	0.005314291
13	4.205	0.321	8.662	0.4935	0.045134566	20.30	0.002223378
14	2.652	0.3602	8.455	0.44	0.06287836	22.29	0.002820922
15	6.992	0.3176	13.88	0.908	0.067380058	28.41	0.002371702
16	3.133	0.4168	15.46	0.5236	0.036143699	13.54	0.002669402
Mean					**0.102387526**	**25.015625**	**0.00396976656**
SD					**0.0862821533**	**10.474853**	**0.00234808473**
Max					**0.366920822**	**49.93**	**0.009981524**
Min					**0.034845036**	**13.17**	**0.001522947**

**Table 3:** Estimated marginal mean and confidence intervals of mean defect depth, maximum defect depth, composite volume lost, area affected and volume lost per unit of area affected.

Estimated Marginal Mean and Confidence Intervals of Mean Defect Depth (µm)		
Air powder	EMM	CI 95%
Sodium bicarbonate	3.68	3.13–4.22
Glycine	2.66	2.11–3.20
Erythritol	3.81	3.26–4.35
**Estimated marginal mean and confidence intervals of maximum defect depth (µm)**		
**Air powder**	**EMM**	**CI 95%**
Sodium bicarbonate	11.63	9.49–13.77
Glycine	6.43	4.29–8.56
Erythritol	9.04	6.90–11.18
**Estimated marginal mean and confidence intervals of volume lost (mm^3^)**		
**Air powder**	**EMM**	**CI 95%**
Sodium bicarbonate	0.09	0.07–0.11
Glycine	0.05	0.03–0.08
Erythritol	0.08	0.06–0.11
**Estimated marginal mean and confidence intervals of area affected (mm^2^)**		
**Air powder**	**EMM**	**CI 95%**
Sodium bicarbonate	25.36	19.76–30.96
Glycine	22.17	16.57–27.77
Erythritol	24.43	18.83–30.04
**Estimated marginal mean and confidence intervals for the volume lost per unit of area affected (mm^3^/mm^2^) × 10^−3^**		
**Air powder**	**EMM**	**CI 95%**
Sodium bicarbonate	3.62	3.11–4.13
Glycine	2.35	1.84–2.86
Erythritol	3.20	2.69–3.71

**Table 4:** Comparative analysis across air powders.

Mean Depth					
Contrast	Estimate, µm	SE, µm	z	p	d
Sodium bicarbonate—Glycine	1.02	0.39	2.60	**0.028**	0.85
Sodium bicarbonate—Erythritol	−0.13	0.39	−0.33	0.983	−0.11
Glycine—Erythritol	−1.15	0.39	−2.93	**0.010**	−0.96
**Maximum Depth**					
**Contrast**	**Estimate, µm**	**SE, µm**	**z**	**p**	**d**
Sodium bicarbonate—Glycine	5.20	1.54	3.37	**0.002**	1.07
Sodium bicarbonate—Erythritol	2.59	1.54	1.68	0.254	0.54
Glycine—Erythritol	−2.61	1.54	−1.69	0.247	−0.54
**Volume**					
**Contrast**	**Estimate, mm^3^**	**SE, mm^3^**	**z**	**p**	**d**
Sodium bicarbonate—Glycine	0.04	0.02	2.26	0.069	0.59
Sodium bicarbonate—Erythritol	0.01	0.02	0.40	0.970	0.10
Glycine—Erythritol	−0.03	0.02	−1.86	0.175	−0.49
**Area Affected**					
**Contrast**	**Estimate, mm^2^**	**SE, mm^2^**	**z**	**p**	**d**
Sodium bicarbonate—Glycine	3.19	4.04	0.79	0.814	0.29
Sodium bicarbonate—Erythritol	0.92	4.04	0.23	0.994	0.08
Glycine—Erythritol	−2.27	4.04	−0.56	0.923	−0.21
**Volume Lost Per Unit of Area Affected**					
**Contrast**	**Estimate, [(mm^3^/mm^2^) × 10^−3^]**	**SE,** **[(mm^3^/mm^2^) × 10^−3^]**	**z**	**p**	**d**
Sodium bicarbonate—Glycine	1.26	0.37	3.43	0.001	0.73
Sodium bicarbonate—Erythritol	0.41	0.37	1.12	0.598	0.24
Glycine—Erythritol	−0.85	0.37	−2.31	0.061	−0.49

Estimate—result derived from contrast analysis; SE—the standard error; z—the z-test statistic; and d—Cohen’s d effect size.

**Table 5:** Particle descriptors for powders E, G and SB.

Powder	N	ECD Mean ± SD (µm)	ECD Min–Max (µm)	ECD D10/D50/D90 (µm)	Aspect Ratio Median [Q1–Q3]	Circularity Median [Q1–Q3]	Circularity Min–Max
E	1600	16.86 ± 7.23	0.64–51.31	8.98/15.64/26.73	1.439 [1.290–1.683]	0.699 [0.605–0.782]	0.129–0.967
G	1600	25.22 ± 11.60	0.64–79.90	11.39/24.55/39.64	1.551 [1.384–1.818]	0.650 [0.564–0.729]	0.050–0.948
SB	1600	23.65 ± 12.42	0.64–95.79	10.87/22.15/37.51	3.333 [2.458–5.104]	0.625 [0.534–0.703]	0.038–0.920

**Table 6:** Previous studies on effect of air-polishing on composite restorations (from oldest to most recent).

Author, Year, [Ref. No]	Air Powders Used	Composite Material Analyzed	Methods	Main Findings
Pelka et al., 2010 [11]	Two sodium bicarbonate powders (no particle size provided) and glycine powder	Nanofilled hybrid composite (Tetric Evo Ceram), two nanofilled flowable composites (Tetric Flow, Grandio Flow)	Profilometric scanning to assess depths and volumes	Air-polishing resulted in substance loss. Defect depth and volume loss were material-dependent, with flowable composites experiencing the greatest damage. Glycine powder caused the least damage to all materials tested.
Salerno et al., 2010 [13]	Sodium bicarbonate and glycine	Light-curing, universal hybrid composite Venus Diamond (Kulzer)	Atomic force microscopy, fractal analysis	Glycine was producing the least surface roughening, correlated with the disappearance of the surface fractal character.
Güler et al., 2011 [23]	Sodium bicarbonate versus prophylaxis powder of calcium phosphate and colloidal anhydrous silica in addition to sodium bicarbonate	Reinforced nanofill composite (Aelite Esthetic Enamel), Silorane resin (Filtek Silorane), microhybrid/hybrid (Filtek Z250), microhybrid/hybrid (Quixfil), nanohybrid (CeramX mono), nanohybrid (Grandio), microhybrid/hybrid (IntenS)	Contact profilometry, SEM	Air-polishing increased the surface roughness of all of the composite resin restorative materials tested. Surface roughening is material-dependent in respect to the composite and powder. Sodium bicarbonate was associated with lower Ra values than the latter powder.
Giacomelli et al., 2011 [12]	Glycine powder sodium bicarbonate powder	Nanohybrid composite resin (Venus Diamond, Heraeus Kulzer)	Atomic force microscopy	Air-polishing caused surface damage. Glycine causes less surface damage than sodium bicarbonate
Barnes et al., 2014 [31]	Sodium bicarbonate 74 µm, Aluminum trihydroxide 80–325 µm calcium carbonate 55 µm, glycine 20–25 µm, calcium sodium phosphosilicate (novamin) 25–120 µm	Light cure hybrid composite (Point 4, Kerr)	Contact profilometry, analysis of epoxy replicas in SEM	No statistically significant difference between the change in surface characterization produced by glycine and sodium bicarbonate. Calcium carbonate, aluminum trihydroxide and sodium bicarbonate powders produced significantly statistically greater changes in surface abrasion of the hybrid composite than glycine or sodium bicarbonate. Calcium sodium phosphosilicate produced the greatest amount of abrasion. Glycine produced the smoothest surfaces.
Janiszewska-Olszowska et al., 2020 [19]	Bicarbonate 40 µm, glicyne 25 µm and erythritol 14 µm	Hybrid composite (Charisma)	3D surface Roughness assessment under confocal microscopy	Sodium bicarbonate had a stronger detrimental effect on composite surface than glycine or erythritol. No advantage of erythritol compared to glycine could be found.
Reinhart et al., 2022 [20]	Erythritol compared with perlite prophy paste in simulated use for 10 years	Nanocomposite (Ceram X Spectra TM ST, Dentsply)	3D surface roughness and abrasion assessment under confocal microscopy following a simulated 10-year interval of prophylactic maintenance	The use of erythritol powder resulted in lower abrasion and roughness compared to Cleanic Prophy Paste (Kerr, Kloten, Switzerland) applied for 200 s with a force of 1.5 N, set with a spring balance.
Németh et al., 2022 [25]	calcium carbonate 54 μm prophylactic powder (Mohs Hardness Index: 3	Nanofill (Filtek Ultimate) and microhybrid (Enamel Plus HRi)	SEM analysis, surface topography analysis in atomic force microscopy synchronized with an Olympus epifluorescence microscope	Air-polishing resulted in increased surface roughness. Disintegrated matrix and free filler particles were visible.

## Data Availability

The original contributions presented in this study are included in the article. Further inquiries can be directed to the corresponding author.
